# The Effect of Wildfire Exposure: Neurological Outcomes, Mental Health, and Epigenetic Insights

**DOI:** 10.3390/genes17040420

**Published:** 2026-04-01

**Authors:** Haneen Abou El Khair, Venika Toor, Lei Cao-Lei

**Affiliations:** Faculty of Health Sciences, University of Ottawa, Ottawa, ON K1S 5S9, Canada; habou024@uottawa.ca (H.A.E.K.); vtoor034@uottawa.ca (V.T.)

**Keywords:** wildfire, mental health, epigenetics, particulate matter (PM_2.5_), environmental exposure, neurotoxicity

## Abstract

**Background/Objectives**: Wildfires are increasing in frequency and intensity worldwide, leading to widespread exposure to wildfire smoke and associated environmental stressors. While the respiratory and cardiovascular effects of wildfire smoke are well established, the potential neurological and mental health consequences have received growing attention. This narrative review synthesizes evidence from animal and human studies examining the effects of wildfire exposure on neurological function, behavior, and mental health, and explores the potential role of epigenetic mechanisms. **Methods:** A structured literature search was conducted using PubMed to identify original research articles examining wildfire exposure in relation to neurological, behavioral, mental health, or epigenetic outcomes. Both human and animal studies were included. **Results:** Experimental animal studies suggest that wildfire smoke exposure can induce neuroinflammation, blood–brain barrier disruption, metabolic alterations, and behavioral changes. Human studies conducted in wildfire-affected populations frequently report an elevated prevalence of depression, anxiety, post-traumatic stress disorder (PTSD), and sleep disturbances. However, many of these studies reflect mental health outcomes associated with wildfire disaster exposure, including evacuation and psychosocial stress, whereas only a subset of studies quantify wildfire smoke or PM_2.5_ exposure. Emerging evidence from both animal models and human studies indicates that wildfire exposure may be associated with changes in epigenetic regulation, including alterations in DNA methylation and miRNA expression. **Conclusions:** Current evidence suggests that wildfire exposure may influence neurological and mental health outcomes through biological and psychosocial pathways. However, the literature remains heterogeneous, and the independent effects of wildfire smoke exposure are often difficult to disentangle from disaster-related stressors. In addition, human evidence linking wildfire exposure to epigenetic changes remains limited, restricting causal inference. Further longitudinal and mechanistic studies integrating exposure assessment, neurological outcomes, and molecular profiling are needed to clarify these relationships.

## 1. Introduction

Wildfires have become an increasingly frequent and severe environmental hazard worldwide, driven by climate change, prolonged drought, and rising global temperatures. In recent decades, many regions—including North America, Australia, and Southern Europe—have experienced unprecedented wildfire seasons, resulting in extensive ecological damage and widespread exposure of populations to wildfire smoke. In Canada alone, the 2023 wildfire season burned approximately 4% of the country’s forest area, representing the largest area burned in recorded history [1]. Importantly, wildfire smoke can travel long distances and affect populations located hundreds or even thousands of kilometers away from the fire source, thereby exposing large populations to elevated levels of air pollutants [2].

Wildfire smoke is a complex mixture of particulate matter (PM) and gaseous pollutants generated during the incomplete combustion of biomass. Its composition typically includes PM_2.5_, carbon monoxide (CO), nitrogen oxides (NO_X_), ozone (O_3_), volatile organic compounds (VOCs), polycyclic aromatic hydrocarbons (PAHs), and trace metals [3]. Its composition varies depending on factors such as vegetation type, combustion efficiency, and atmospheric conditions. Among these components, PM_2.5_ has received particular attention due to its ability to penetrate deep into the respiratory system and enter the systemic circulation. Compared with urban air pollution derived primarily from traffic or industrial sources, wildfire smoke contains a distinct mixture of combustion-derived particles and organic compounds that may produce different toxicological effects. Consequently, wildfire smoke exposure is increasingly recognized as a unique environmental exposure requiring dedicated investigation.

In addition to the toxicological effects of smoke inhalation, wildfire events also represent complex environmental disasters that may generate substantial psychological stress through evacuation, displacement, property loss, and threat to life [4]. As a result, research on wildfire-related health effects often reflects two partially overlapping pathways of exposure. The first pathway involves direct inhalation of wildfire smoke and air pollutants, which may influence neurological and cognitive outcomes through biological mechanisms such as neuroinflammation, oxidative stress, and vascular dysfunction. The second pathway reflects disaster-related psychosocial stress, which can contribute to mental health outcomes, including depression, anxiety, and post-traumatic stress disorder (PTSD). Distinguishing these pathways is important for interpreting the literature, as many epidemiological studies capture disaster-related experiences rather than isolated smoke exposure.

While the respiratory and cardiovascular effects of wildfire smoke have been widely documented [3], increasing attention has focused on its potential neurological and mental health consequences. Experimental studies suggest that inhaled PM may affect the central nervous system through several mechanisms, including systemic inflammation, disruption of the blood–brain barrier, microglial activation, oxidative stress, and altered neurotransmission. These biological responses have been associated with changes in cognition, behavior, and emotional regulation in animal models [5,6,7,8]. In human populations, wildfire events have been associated with increased prevalence of depression, anxiety, PTSD, sleep disturbances, and, in some studies, cognitive impairments [4,9,10,11,12,13]. However, the relative contributions of toxicological smoke exposure versus disaster-related stress remain an area of active investigation.

Epigenetic mechanisms may provide an additional biological framework linking environmental exposures to long-term neurological and mental health outcomes. Epigenetic mechanisms—including DNA methylation, histone modifications, and non-coding RNA—can modify gene expression without altering the underlying DNA sequence and may respond dynamically to environmental stimuli such as air pollution. Emerging evidence from experimental models suggests that wildfire smoke exposure may alter gene expression patterns and DNA methylation profiles in both somatic and germline tissues [14,15,16]. However, human evidence remains limited, and the extent to which such epigenetic alterations contribute to mental health outcomes following wildfire exposure is not yet well understood.

Given the rapidly increasing frequency and intensity of wildfires globally, synthesizing current evidence on the neurological and mental health consequences of wildfire exposure is of growing public health importance. The conceptual framework guiding this review considers two partially overlapping pathways through which wildfires may influence neurological and mental health outcomes: (1) toxicological exposure to wildfire smoke and air pollutants, and (2) psychological stress associated with wildfire disasters, including evacuation, displacement, and perceived threat. Experimental animal studies provide mechanistic insight into biological responses to smoke exposure, whereas human studies more often capture mental health outcomes associated with wildfire events and disaster-related experiences. Epigenetic mechanisms may represent potential pathways linking environmental exposures to longer-term neurological and psychological effects.

This narrative review, therefore, integrates findings from both experimental animal studies and human investigations examining the neurological, cognitive, and mental health effects of wildfire exposure, with particular attention to emerging evidence regarding epigenetic mechanisms. To improve conceptual clarity, we distinguish between studies examining wildfire smoke exposure and those assessing mental health outcomes in wildfire disaster contexts. Specifically, we (1) summarize experimental animal studies investigating neurobiological effects of wildfire smoke exposure, (2) review human evidence on mental health and cognitive outcomes associated with wildfire events and smoke exposure, and (3) examine current evidence on epigenetic changes associated with wildfire exposure across species. Finally, we discuss potential mechanistic pathways, key limitations of the current literature, and priorities for future research.

## 2. Materials and Methods

This study was conducted as a narrative review intended to synthesize current evidence on the neurological, mental health, and epigenetic effects of wildfire exposure. A structured literature search was performed using PubMed to identify relevant peer-reviewed articles. The search included studies published from database inception to March 2026. Only articles published in English were considered.

Search terms included combinations of keywords related to wildfires, wildfire smoke, PM_2.5_, neurological outcomes, mental health, behavior, and epigenetic mechanisms. Titles and abstracts were screened to identify studies examining wildfire exposure in relation to neurological, behavioral, mental health, or epigenetic outcomes. Potentially relevant articles were then assessed through full-text review.

Because the objective of this review was to provide a narrative synthesis of the literature, a formal risk-of-bias assessment was not performed. Instead, findings from eligible studies were qualitatively summarized to highlight key mechanistic insights, epidemiological associations, and emerging research gaps.

### 2.1. Human Studies: Mental Health Outcomes

To identify studies examining mental health outcomes following wildfire exposure in humans, PubMed was searched using the following terms:

(Wildfire* OR Bushfire* OR Forest fire*) AND (“Mental health” OR Mental Illness OR Psychological Disorder* OR Psychiatric Disorder* OR PTSD OR “Emotional Well-being” OR Anxiety OR Depression).

The search yielded 523 records. After removing duplicates and screening titles and abstracts, studies were excluded if they did not focus on wildfire-related environmental exposure, did not assess psychological outcomes, were not original research articles, or did not report quantified mental health measures. Two additional relevant studies were identified through backward citation tracking of included articles. Following screening, 25 studies met the inclusion criteria and were included in the review.

### 2.2. Animal Studies: Neurological and Behavioral Outcomes

To identify animal studies investigating the neurological and behavioral effects of wildfire exposure, PubMed was searched using:

(Wildfire* OR Bushfire* OR Forest fire* OR “wildfire smoke”) AND (brain* OR neuro* OR cognition OR learning* OR memory* OR behavior*)*.*

This search yielded 270 records. Studies were excluded if exposures were not attributable to wildfire smoke (e.g., non-wildfire particulate matter) or if wildfire exposure was not adequately characterized. For this review, adequately characterized exposure referred to studies that reported wildfire-related smoke exposure through controlled experimental exposure to biomass or wildfire smoke, or through environmental indicators such as measured or modeled wildfire-related particulate matter (e.g., PM_2.5_) concentrations. Studies were also excluded if outcomes were not neurological, behavioral, or epigenetic in nature. The PubMed “other species” filter was applied to restrict results to animal models. Following screening, seven animal studies met the inclusion criteria and were included.

### 2.3. Human Epigenetic Studies

To identify human studies examining epigenetic outcomes associated with wildfire exposure, PubMed was searched using:

(Wildfire* OR Bushfire* OR Forest fire*) AND (Epigenetic OR “DNA methylation” OR RNA OR Epigenomic OR “Histone modification” OR “Epigenetics, Genetic”)*.*

This search yielded 103 records. Studies were excluded if they did not focus on wildfire-related environmental exposure, did not assess epigenetic mechanisms, or did not report relevant biological outcomes. One human study met the inclusion criteria. One additional relevant study was identified through backward citation tracking and included, resulting in a total of two human epigenetic studies.

### 2.4. Animal Molecular and Epigenetic Studies

Animal studies investigating molecular or epigenetic mechanisms were identified using PubMed with the following query:

(Wildfire* OR Bushfire* OR Forest fire*) AND (“Molecular Mechanism*” OR Epigenetic* OR “DNA Methylation” OR “Gene Expression” OR “Histone Modification” OR “RNA Expression”)*.*

This search yielded 28 records. Studies were excluded if they were duplicates, did not report brain-related outcomes, involved exposures not attributable to wildfire smoke, or used non-rodent animal models considered less relevant for translational interpretation. The PubMed “other species” filter was applied to restrict results to animal models. One study met the inclusion criteria. Due to the limited number of eligible studies, three additional relevant studies were identified through backward citation tracking of included articles. As a result, a total of four animal epigenetic studies were included.

## 3. Results

The results are organized to summarize evidence from experimental animal studies and human investigations examining the neurological, psychological, and epigenetic effects of wildfire exposure. First, findings from animal models are presented to highlight mechanistic pathways, including neuroinflammation, metabolic disruption, behavioral alterations, and intergenerational effects. Next, evidence from human studies is summarized according to major mental health outcomes associated with wildfire exposure. Finally, emerging evidence on epigenetic modifications related to wildfire exposure is reviewed in both animal models and human populations. An overview of the main findings from the included studies is provided in Table 1 and Table 2.

### 3.1. Experimental Animal Evidence

Investigating the specific health effects of wildfire smoke under controlled conditions presents logistical challenges. Consequently, animal studies often employ models that use proxies for wildfire smoke, such as smoke from controlled wood or biomass combustion. These models are designed to replicate key features of real-world wildfire smoke, including its complex particulate and chemical composition (e.g., PM_2.5_, PAHs, VOCs) [3], and are therefore treated as experimental surrogates for understanding the biological effects of wildfire exposure.

#### 3.1.1. Neuroinflammatory Responses, Metabolic and Neurobehavioral Alterations

Animal studies suggest that wildfire smoke exposure can induce central nervous system (CNS) inflammation, disrupt the blood–brain barrier (BBB), and activate glial cells. In mice exposed to PM_2.5_ derived from real-world wildfire smoke, investigators observed BBB disruption within the neurovascular unit, accompanied by astrocytosis, microgliosis, and infiltration of peripheral immune cells, supporting a neuroinflammatory response [17]. Elevated intracellular proinflammatory markers, such as inducible nitric oxide synthase (iNOS), were also detected in CNS microglia. Endothelial analysis identified platelet endothelial cell adhesion molecule-1 (CD31)-high cells linked to vascular repair and CD31-medium cells with increased inflammatory markers. Metabolomic profiling further indicated reductions in several neuroprotective metabolites that are involved in neuronal energy metabolism and aging-related pathways [17].

A separate study examined the temporal dynamics of these responses using a model of intermittent woodsmoke exposure [8]. This study reported dynamic shifts in endothelial and immune cell populations. Metabolomic analyses indicated a broader disruption of neurometabolic pathways, including changes in metabolites related to energy metabolism, amino acid pathways, and neuroactive signaling molecules [8]. These findings suggest that wildfire smoke exposure may induce both inflammatory and metabolic perturbations in the brain that evolve following exposure.

Evidence for longer-lasting neurobehavioral consequences was provided by a sub-acute woodsmoke exposure study in female mice [18]. Metabolomic analysis of the prefrontal cortex revealed alterations in metabolites associated with neuronal energy metabolism and neurotransmitter pathways, including reductions in nicotinamide adenine dinucleotide (NAD^+^) and serotonin following exposure [18]. Behavioral testing demonstrated increased immobility in forced-swim assays, consistent with depression-like behavior, while grip strength remained unchanged. These metabolic alterations may reflect disruptions in neurochemical pathways involved in mood regulation and neuronal function. Neuroinflammatory profiling further showed elevated hippocampal NLRP3 inflammasome and caspase-1 protein levels several weeks after exposure, suggesting persistent inflammatory activation despite no alteration in measured cytokines. Overall, the metabolic changes observed following woodsmoke exposure were modest compared with those associated with natural aging. Among post-exposure interventions, combined resveratrol and nicotinamide mononucleotide (RNMN) was the most effective at mitigating wildfire smoke–induced metabolic disturbances.

**Table 1:** Summary of Neurological and Epigenetic Effects of Wildfire Smoke Exposure in Animal Models.

Study (y)	Species	Total Sample Size	Exposure	Sample Source (s)	Epigenetic Mechanism	Main Findings
Neurological and Behavioral Studies						
Capitanio et al. (2022) [7]	Infant rhesus monkeys in the first third of gestation	89	Outdoor-housed rhesus monkeys exposed to Camp Fire wildfire smoke with elevated PM_2.5_ levels	Blood samples	–	Elevated plasma CRP, blunted cortisol response, more passivity, and memory impairment compared with animals conceived after the smoke had dissipated
Sosedova et al. (2020) [6]	3-months-old outbred albino rats (10 males and 60 females of parental generation; 80 males and 80 females of offspring)	230	Paternal whole-body biomass smoke inhalation for 4 weeks (5 d/week, 4 h/day) with controlled CO, PM_2.5_, and aldehyde concentrations	–	–	Paternal exposure led to reduced locomotion and increased anxiety-like behavior in immediate post-exposure offspring; decreased exploration in females; impaired spatial memory in males (↑ Morris water maze latency)
Wardhani et al. (2024) [19]	Male and female C57BL/6 mice, as well as female Sham and ovariectomized (OVX) mice at 6–8 weeks of age	64	Whole-body inhalation of woodsmoke (4 h/day × 2 days)	Brain tissue and plasma samples	–	Sex- and hormone-dependent neuroinflammatory responses: males showed upregulation of IL-1β, CXCL1, TGF-β, and IL-6 mRNA expression, along with increased cortical GFAP expression (astrogliosis); OVX females exhibited heightened inflammation (↑ CCL2, CXCL1 in the cortex; ↓ TGF-β in the hippocampus)
Scieszka et al. (2022) [17]	2-months-old male C57BL/6 mice	24	WFPM exposure in mobile lab (300 km from 2020 Western US fires); 4 h/day × 20 days; average PM_2.5_ 104 μg/m^3^	Bronchoalveolar lavage fluid, bone marrow cells, brain tissue, blood- derived cells	–	Increased macrophages and endothelial activation (↑ VCAM-1, ICAM-1), elevated Aβ_42_ levels, microglial activation (↑ CCL2, iNOS), and reduced NAD^+^, NADH, taurine, and succinate
Scieszka et al. (2023) [8]	8-weeks-old female C57BL/6 mice	60	Whole-body woodsmoke exposure for 4 h every other day × 14 days	Hippocampus, brain tissue, bronchoalveolar lavage fluid	–	Persistent hippocampal neuroinflammation: early CD31^Hi^ endothelial surge at Day 14, prolonged microglial activation to Day 28; sustained metabolic disruptions, including ↓ NAD^+^, ↓ glutamate, ↑ α-Aminoadipic acid, ↓ 5α-DHP, and other altered metabolites
Scieszka et al. (2025) [18]	18-months-old female C57BL/6J mice	60	Whole-body woodsmoke inhalation at an average PM ≈ 448 µg/m^3^	Pre-frontal cortex (PFC) and hippocampus	–	Persistent PFC NAD^+^ and serotonin depletion, depression-like behavior, and elevated hippocampal NLRP3/caspase-1; RNMN partially restored metabolites and neurotransmitters, DQ modestly effective, RNDQ increased aging-related metabolites
Gorgun et al. (2017) [5]	CB57BL/6 male mice	Initially 18 sham and ~18 smoke-exposed; final n varied by test (Rotarod n = 8/group, Fear Conditioning n = 10, Open Field n = 12–15)	Smoke generated from smoldering aspen wood shavings, 60-min exposure with 10–20 s venting intervals	Brain tissue	–	8 months post-exposure, mice exhibited hippocampal astrogliosis, microgliosis, demyelination, elevated pro-inflammatory markers (e.g., IL-1β, TNF-α, IFN-γ, CCL2), and stress-related genes (e.g., *c-Fos*, *HMOX1*), and persistent anxiety-like behavior
Molecular and Epigenetic Mechanisms						
Schuller et al. (2021) [14]	8-week-old male Apoe−/− mice on a C57BL/6 background	20	Whole-body smoke exposure for 2 h/day, 5 d/week × 40 days	Sperm	Reduced representation bisulfite sequencing (RRBS)	Differential sperm DNA methylation, with 3353 DMRs identified (703 hypomethylated, 2650 hypermethylated)
Schuller et al. 2024 [16]	8-week-old male Apoe−/− mice on a C57BL/6 background	16	Simulated wildfire smoke exposure (2 h/day, 5 days/week for 16 weeks) at average 39 ± 13 mg/m^3^ PM	Prefrontal cortex (PFC)	RNA sequencing (Illumina HiSeq)	2862 differentially expressed genes (1396 upregulated, 1466 downregulated), including increases in *Vcl*, *Adamts3*, *Pcdhgc3* and reductions in *Psg16*, *Apoo* genes; serotonergic, cholinergic, and dopaminergic pathways were affected
Brown et al. 2022 [15]	Adult female rhesus macaques (Macaca mulatta)	22	Early-life wildfire smoke exposure to high-PM_2.5_/ozone levels (0–3 months) vs. low-exposure controls	Nasal epithelium samples and peripheral blood	RNA sequencing (Illumina NextSeq 550); whole-genome bisulfite sequencing (Illumina NovaSeq 6000)	3370 differentially methylated regions (DMRs) (86% hypermethylated); DMRs enriched in neuronal and immune-related pathways, including IL-15 production and Th1/Th2 activation, among others; the *FLOT2* gene showed persistent differential expression
Vokina et al. (2021) [20]	3-months-old outbred albino Wistar male rats	40	Peat smoke inhalation for 24 h	Blood cells	Enzyme-modified comet assay (DNA methylation; HpaII/MspI)	Reduced global DNA methylation without DNA fragmentation; increased anxiety-like behavior; no Morris water maze deficits; EEG showed increased right-hemisphere δ and reduced left-hemisphere β2 activity

Aβ_42_ = Amyloid-β_42_; *Adamts3* = Thrombospondin Type 1 Motif 3; *Apoo* = Apolipoprotein O; β2 = Beta-2 frequency band; CCL2 = Chemokine C-C Motif Ligand 2; CO = Carbon Monoxide; CRP = C-Reactive Protein; CD31^Hi^ = High-Expressing Platelet Endothelial Cell Adhesion Molecule-1 (PECAM-1); CXCL1 = Chemokine (C-X-C motif) Ligand 1; 5α-DHP = 5α-Dihydroprogesterone; DQ = Dasatinib + Quercetin treatment; δ = Delta frequency band; EEG = Electroencephalography; *FLOT2 *= Flotillin 2 gene; GFAP = Glial Fibrillary Acidic Protein; *HMOX1 *= Heme Oxygenase 1 gene; HpaII/MspI = Restriction enzymes for DNA methylation analysis; IL-1β = Interleukin-1 Beta; IFN-γ = Interferon Gamma; ICAM-1 = Intercellular Adhesion Molecule-1; iNOS = Inducible Nitric Oxide Synthase; IL-6 = Interleukin-6; IL-15 = Interleukin-15; NAD^+^ = Nicotinamide Adenine Dinucleotide; NADH = Nicotinamide Adenine Dinucleotide (reduced); PM_2.5_ = Particulate Matter ≤ 2.5 µm in aerodynamic diameter; *Pcdhgc* = Protocadherin Gamma Subfamily C 3; *Psg16* = Pregnancy Specific Glycoprotein 16; RNMN = Resveratrol + Nicotinamide Mononucleotide treatment; RNDQ = Resveratrol + Nicotinamide Mononucleotide plus Dasatinib + Quercetin treatment; TNF-α = Tumor Necrosis Factor-alpha; TGF-β = Transforming Growth Factor-Beta; Th1/Th2 activation = Activation of T-helper 1 and T-helper 2 cells; VCAM-1 = Vascular Cell Adhesion Molecule-1; *Vcl* = Vinculin; WFPM = Wildfire-smoke-derived PM_2.5_; ↑ = increased; ↓ = decreased.

**Table 2:** Summary of the Effects of Wildfires on Humans.

Author	Sample Group	Exposure	Exposure Type	Assessment	Sample	Epigenetic Mechanism	Findings
Belleville et al. (2019) [21]	394 Participants (≥18 y)	2016 McMurray Wildfires (Canada)	Disaster	PCL-5, ISI, PSQI-A, PTCI, WCQ	–	–	PTSD symptoms (PCL-5 ≥ 33): 62.5% PTSD diagnosis: 29.1% MDD diagnosis: 25.5% High levels of depression, insomnia, and trauma-related sleep disturbances
Belleville et al. (2021) [9]	1510 participants (838 Female; 672 Male)	1 year after the 2016 Fort McMurray Wildfires (Canada)	Disaster	PCL-5, ISI, PHQ-9, GAD-7, CAGE substance abuse screening tool	–	–	Insomnia was the most common diagnosis post-wildfire (28.5%). 38% had ≥1 probable mental health diagnosis one-year post-wildfire Pre-wildfire depression increased the risk of PTSD 5× 87.1% with probable PTSD had ≥1 comorbid diagnosis
Brown et al. (2019) [10]	5866 Grade 7–12 students	2016 Wildfire in Fort McMurray (Canada)	Disaster	PHQ-A, HADS, CRAFFT, Tobacco use questionnaire, Rosenberg, Kidscreen-10	–	–	Self-esteem and quality of life scores were ↓ in Red Deer. HADS anxiety score ↑ in Red Deer Depression severity, suicidal ideation, and tobacco use were ↑ in Fort McMurray
Bryant et al. (2018) [22]	1017 Participants	2009 Black Saturday Wildfire in Victoria (Australia)	Disaster	PCL-S, PHQ-9, K6, AUDIT-C	–	–	Higher Probable PTSD and major depression, and lower resilience in high-impacted communities Mental health problems decreased over time
Hong et al. (2022) [23]	315 Participants (≥19 y)	2019 wildfires in Gangwon (South Korea)	Disaster	Post-disaster psychological responses using a checklist & CGI-S	–	–	Participants reported experiencing insomnia, anxiety, chest tightness, grief, flashbacks, and depression Symptom severity (CGI-S) decreased within 1 month and remained stable at 6 months
Isaac et al. (2023) [24]	126 Participants	Wildfires in Australia, USA, Canada	Disaster	DDNSI, GAD-7, ISI, PHQ-9, PSQI, PCL-5	–	–	U.S. participants showed higher rates of severe anxiety and greater insomnia, depression, and trauma-related symptoms Canadian participants reported more nightmares than Australian and U.S. participants
Mao et al. (2022) [25]	186 Participants	5 years after the 2016 Fort McMurray Wildfire (Canada)	Disaster	PHQ-9, PCL-C	–	–	The prevalence of MDD was 45%, and PTSD was 39.6% Past diagnosis of depression before the wildfire increased the risk of PTSD by 5 times and predicted the likelihood
McFarlane et al. (1997) [26]	1974 Households 100 non-bushfire participants	Ash Wednesday Bushfire (Australia)	Disaster	GHQ	–	–	Disaster victims showed higher psychiatric morbidity (women 19–36%, men 13–31%), double that of controls at 12 months
	80 Participants			GHQ and DIS			23% scored >4 at 20 months, 20 months after the bushfire 6 people were diagnosed as currently having a disorder, 5 received a diagnosis of PTSD, 3 received a diagnosis of depressive disorder
Mellish et al. (2024) [27]	318 Participants	2019/2020 Bushfires (Australia)	Disaster	IES-R, Brief-COPE, PTGI	–	–	25.5% had a probable PTSD diagnosis Participants who experienced Indirect exposures had maladaptive coping mechanisms and lower posttraumatic growth
Psarros et al. (2018) [28]	102 Male firefighters	2007 Wildfires in Pyrgos, Olympia (Greece)	Disaster	SCL-90, AIS	–	–	Permanent firefighters had lower PTSD risk than seasonal workers Higher neuroticism, anxiety, depression, and fear of imminent death at work increased PTSD risk
Ritchie et al. (2021) [29]	329 Participants	2016 Wildfire in Fort McMurray (Canada)	Disaster	PHQ-9, GAD-7, PCL-5	–	–	Prevalence post-wildfire: MDD 23.4%, GAD 18.7%, PTSD 11% (higher in females)
Silveira et al. (2021) [12]	725 Participants (18–84 y)	2018 California Camp Fire (USA)	Disaster	PCL-5, GAD-7, PHQ-9	–	–	Direct wildfire exposure, prior childhood trauma, and sleep disturbances increased PTSD, depression, and anxiety Self-reported resilience is inversely associated with anxiety, depression and PTSD
Yelland et al. (2010) [30]	159 Participants (8–18 y)	2005 Bushfires in Lower Eyre Peninsula (Australia)	Disaster	PTSD-RI-R	–	–	PTSD symptoms were generally mild, with 17% moderate and 10% severe, more than a year post-bushfire Younger children showed higher symptom severity
Lee et al. (2025) [31]	739 Participants	2025 Wildfires in Los Angeles (USA)	Disaster	CES-D-10, GAD-7, PC-PTSD-5	–	–	Participants who evacuated had a higher risk of depression and PTSD No significant difference in evacuation status for anxiety
Oliveira et al. (2023) [32]	139 Firefighters	2017 Wildfires in Portugal	Disaster	PCL-5, BSI, QEPAT	–	–	13.8% of participants had possible psychopathology, and 9.2% satisfied criteria for PTSD PTSD scores were increased with risk factors such as paranoid ideation, hostility, depression, anxiety, and phobic anxiety
Mao et al. (2024) [33]	298 Participants	2023 Wildfires in Alberta and Nova Scotia (Canada)	Disaster	PHQ-9	–	–	Prevalence of MDD was 56.1% in disaster impacted areas Compared with employed participants, those who were unemployed were twice as likely to report moderate to severe MDD
Moosavi et al. (2019) [34]	290 Participants	2016 Wildfires in Fort McMurray (Canada)	Disaster	DSM, PHQ-9, GAD-7, PCL-5, DUDIT, AUDIT	–	–	Higher prevalence rates of PTSD, GAD and MDD for 1 month PTSD and MDD prevalence rates were higher after 6 months when compared with the general population Previous history of depressive disorder predicted the likelihood of developing MDD in the general population 6 months after the wildfire
Agyapong et al. (2018) [13]	486 Participants	2016 Wildfires in Fort McMurray (Canada)	Disaster	GAD-7, DUDIT, AUDIT, Fagerstrom Test for Nicotine Dependence	–	–	GAD prevalence 6 months post-wildfire: 19.8% Participants with previous anxiety disorders were 7 times more likely to have higher GAD symptoms after the wildfire GAD symptoms associated with increased substance use
Toit et al. (2026) [35]	2967 Adolescents	2019–2020 Black Summer bushfires (Australia)	Disaster	PHQ-A, SCAS, DQ5, ISI, SIDAS	–	–	Adolescents exposed to bushfires did not significantly show elevated symptoms 24 months post-bushfire compared to unexposed adolescents Demographic factors are the strongest predictors of mental health outcomes
Papadatou et al. (2012) [4]	1468 Adolescents	6 months after 2007 Wildfire in Peloponnese (Greece)	Disaster	CRIES-13	–	–	Rate of probable PTSD was 29.4% and 20% for probable depression Pre-disaster factors, such as death or illness, were associated with increased PTSD and depression symptoms
Jones et al. (2002) [36]	19 Families	6 months after 1990 Wildfire in California (USA)	Disaster	DIS, CBQ	–	–	High-loss participants reported higher levels of PTSD compared to low-loss participants Parents reported significantly more PTSD symptoms than children
Jung et al. (2025) [37]	86,668 ED visits	2020 California Wildfires (USA)	Wildfire fine particulate matter PM_2.5_	Daily ED visits were counted and identified using ICD	–	–	86,609 mental health-related ED visits were linked to PM_2.5_ exposure Wildfire-specific PM_2.5_ was positively associated with mental health conditions Substance use was the most common adult diagnosis; anxiety was most common among youth and seniors
Zhu et al. (2024) [38]	1,897,865 ED visits	2007–2018 Wildfire Smoke PM_2.5_ (USA)	Wildfire smoke PM_2.5_	Anxiety Disorders classified by ICD	–	–	Wildfire smoke PM_2.5_ is positively associated with ED visits for anxiety disorders An exposure to 10 μg m^−3^ increase in PM_2.5_ smoke in 48 h led to 0.6% increased risk in ED visits for anxiety disorders
Mirabelli et al. (2022) [39]	5946 Participants	2018 Wildfires in Oregon (USA)	Wildfire smoke	BRFSS	–	–	4 weeks of heavy smoke has been linked with a 34% increased prevalence of feeling nervous, anxious, or on edge during the past 2 weeks Heavy and medium smoke exposure increased uncontrolled worrying by 29–30%
Cleland et al. (2022) [11]	10,228 Participants	Daily and Hourly exposure to PM_2.5_ was collected (USA)	Fine particulate matter PM_2.5_	Attention-oriented brain-training game	–	–	PM_2.5_ was negatively associated with the attention score Medium and heavy smoke had a negative association with attention Strongest association with reduced attention within 3 h of exposure to PM_2.5_
Goodrich et al. (2025) [40]	99 Firefighters	10 months after wildfire season in California (USA)	Wildfire Smoke	Responded to ≥1 Wildland–urban interface fire	Blood Samples	DNA methylation: Infium EPIC array Relative Abundance of miRNAs: nCounter Human v3 miRNA expression panel	65 miRNAs were different when compared to post wildfires No significant differences in DNA methylation between pre and post wildfires The miRNA, hsa-miR-518c-3p, was downregulated when exposed to wildfires
Xu et al. (2023) [41]	479 Australian Women	Wildfire PM_2.5_ and Non-wildfire PM_2.5_ (Australia)	Wildfire fine particulate matter PM_2.5_	A 3-year average of wildfire-related and non-wildfire-related PM_2.5_ data based on participant’s location	Whole Blood Sample	DNA methylation: Infinium™ HumanMethylation450 BeadChip array (Illumina 450 k array)	A strong association between 7 measures of global DNA methylation that decreased and wildfire-related PM_2.5_ CpGs and DMRs associated with wildfire-related PM_2.5_ were different from non-wildfire-related PM_2.5_ Hypomethylation of VTRNA2-1

AIS = Athens Insomnia Scale; AUDIT-C = Alcohol Use Disorders Identification Test; BRFSS = Behavioral Risk Factor Surveillance System; BSI = Brief Symptom Inventory questionnaire; CBQ = Children’s Behavioral Questionnaire; CES-D-10 = Center of Epidemiologic Studies Depression Scale, 10-item version; CGI-S = Clinical Global Impression Scale-Severity; CRAFFT = Car, Relax, Alone, Forget, Friends, Trouble screen; CRIES-13 = Children’s Revised Impact of Event Scale; DDNSI = The Disturbing Dream and Nightmare Severity Index; DIS = Diagnostic Interview Schedule; DQ5 = Distress Questionnaire 5; DSM = PTSD Checklists for Diagnostic and Statistical Manual; DUDIT = Drug Use Disorder Identification Test; ED = Emergency Department; GAD-7 = General Anxiety Disorder-7; GAD = General Anxiety Disorder; HADS = Hospital Anxiety and Depression Scale; ICD = International Statistical Classification of Diseases and Related Health Problems; ISI = Insomnia Severity Index; K6 = Kessler Psychological Distress Scale; MDD = Major Depressive Disorder; PCL-5 = Posttraumatic Stress Disorder Checklist for DSM-5; PCL-S = Posttraumatic Stress Disorder Checklist; PC-PTSD-5 = Primary Care PTSD Screen for DSM-5; PHQ-9 = Patient Health Questionnaire-9; PHQ-A = Patient Health Questionnaire: Adolescent Version; PSQI = Pittsburgh Sleep Quality Index; PTCI = The Posttraumatic Cognitions Inventory; PTSD = Post-traumatic Stress Disorder; PTSD-RI-R = Post-traumatic Stress Disorder Reaction Index for Children-Revised; QEPAT = Questionário de Exposição e Perturbação dos Acontecimentos Traumáticos; SCAS = Spence Children’s Anxiety Scale; SCL-90 = Symptom Checklist-90-R; SIDAS = Suicidal Ideation Attributes Scale; VTRNA2-1 = Vault RNA 2-2, pseudogene; WCQ = Ways of Coping Questionnaire; ↑ = increased; ↓ = decreased.

In another study, a single acute exposure to combustion smoke was associated with neuroinflammation persisting eight months later [5]. Mice showed elevated glial fibrillary acidic protein (GFAP) and ionized calcium-binding adapter molecule 1 (Iba-1) expression in the hippocampus, accompanied by reduced myelin basic protein (MBP) levels in the cornu ammonis region 3 (CA3) of the hippocampus, suggesting long-term disruptions in glial function and myelination. Gene expression analyses revealed sustained upregulation of proinflammatory markers and stress-related genes. Interestingly, early alterations in genes related to energy metabolism were no longer detectable at the later time point, suggesting that some metabolic responses may be transient. Behaviorally, exposed animals exhibited heightened anxiety-like behaviors.

Sex-specific responses to wildfire smoke exposure have also been reported. In an acute woodsmoke exposure study, male mice showed increased pro-inflammatory signaling and marked astrogliosis [19]. In contrast, female mice showed region-specific inflammatory responses. When ovariectomized, females exhibited heightened cortical inflammation and suppressed anti-inflammatory signaling in the hippocampus, underscoring the regulatory role of ovarian hormones. Lipidomic analyses further indicated sex-dependent alterations in lipid metabolism pathways following smoke exposure, suggesting that biological sex and hormonal status may influence metabolic responses to wildfire pollutants.

#### 3.1.2. Intergenerational Effects

Prenatal exposure to wildfire smoke may contribute to lasting effects on offspring neurodevelopment and behavior. A study [7] of pregnant female rhesus monkeys and their offspring during the 2018 California wildfire season found that infants exposed in utero, particularly during the first third of gestation, had elevated inflammatory markers (e.g., plasma C-reactive protein) and a lower cortisol response, suggesting disruption of hypothalamic–pituitary–adrenal (HPA) axis function. Behaviorally, these animals exhibited impaired visual recognition memory and increased passivity. Retrospective comparison with a large control cohort confirmed that these changes were not attributable to conception timing alone, reinforcing the developmental impact of wildfire smoke exposure.

Intergenerational effects have also been suggested through paternal exposure models. In a study [6] in which adult male albino rats were exposed to biomass smoke, offspring conceived immediately after exposure exhibited heightened anxiety-like behavior, impaired spatial memory, and reduced exploration, with more pronounced effects observed in female offspring. In contrast, offspring conceived 60 days later showed partial recovery, although some sex-specific anxiety behaviors persisted.

Collectively, animal studies provide converging evidence that wildfire smoke exposure induces neuroinflammation, disrupts blood–brain barrier integrity, alters brain metabolism, and produces behavioral changes. These effects can endure for weeks to months after exposure and are modulated by sex and hormonal status. Importantly, prenatal and preconception exposures suggest that wildfire smoke may also exert intergenerational effects, raising concerns about long-term neurological vulnerability following exposure during sensitive developmental windows.

### 3.2. Human Evidence

#### 3.2.1. Mental Health Outcomes in Wildfire Disaster Contexts

Wildfires represent not only environmental pollution events but also large-scale natural disasters that may produce substantial psychological stress through evacuation, displacement, threat to life, and property loss. As a result, many epidemiological studies examining mental health outcomes following wildfires capture the effects of disaster-related stressors rather than isolated smoke exposure. These psychosocial factors can contribute to increased prevalence of depression, anxiety, PTSD, and sleep disturbances among affected populations.

Following wildfire exposure, individuals frequently report depressive symptoms or meet criteria for major depressive disorder (MDD) [4,12,21,22,23,25,26,29,33]. Studies consistently show that direct exposure is associated with higher depression scores [12], and that communities experiencing greater wildfire impact report a higher prevalence of MDD (10.9%) compared with medium- (4.7%) and low-impact (4.6%) communities [22]. Several individual-level factors further increase vulnerability to MDD and PTSD, including unemployment, use of sleep medication, a prior diagnosis of depression, and willingness to seek mental health support [25]. Unemployed participants were twice as likely to report moderate to severe MDD [33].

In parallel with depressive symptoms, wildfire exposure has been strongly associated with increased prevalence of PTSD [4,9,12,28,29,31,34,36]. The prevalence of PTSD increased to 11% after wildfires [29]. Direct exposure led to greater PTSD scores [12]. Firefighters with permanent jobs had a decreased risk of suffering from PTSD compared to temporary firefighters [28]. Participants with a fear of death during work had an increased risk of developing PTSD [28]. PTSD scores increased with risk factors such as paranoid ideation, hostility, depression, anxiety, and phobic anxiety [32]. Mental health symptom severity is dependent on financial stress and previous mental health conditions [9]. Age and sex differences have also been reported. Younger children tend to exhibit more severe symptoms, although sex differences are inconsistent: some studies report no differences between girls and boys [30]. However, among college students, PTSD prevalence differed by sex, with higher rates observed in females (12.6%) than in males (6.8%) [29]. Parents reported higher levels of PTSD symptoms compared to their children [36]. Community-level exposure remains a critical determinant, with high-impact communities reporting substantially higher rates of probable PTSD (10.9%) than medium- (5.6%) or low-impact (1.9%) communities [22]. After exposure to wildfire, the rates of psychological outcomes such as PTSD decreased over time [22].

Anxiety symptoms are also commonly reported following wildfires [10,13,23,24]. After the 2016 Fort McMurray wildfires, the prevalence of generalized anxiety disorder (GAD) reached 18.7% among college students [29], and anxiety scores increased significantly among students in grades 7–12 [10]. Cross-national comparisons suggest greater anxiety severity among participants in the United States compared with those in Australia and Canada [24].

Sleep disturbances represent another common psychological outcome of wildfire exposure. Insomnia has been reported as one of the most prevalent diagnoses following wildfires, with a reported prevalence of 28.7% [9]. Participants frequently reported repeated disturbed memories (77.4%), emotional distress when reminded of the event (76.7%), and difficulty falling or staying asleep (72.5%) [21]. Individuals exposed to wildfire who also had a history of childhood trauma experienced greater sleep disturbance and were at increased risk for anxiety, depression, and PTSD [12].

Despite these adverse outcomes, resilience has emerged as an important moderating factor. Self-reported resilience following wildfire exposure has been inversely associated with symptoms of anxiety, depression, and PTSD [12]. However, resilience levels vary by exposure intensity, with heavily affected communities reporting lower resilience (76.8%) than medium- (90.6%) or low-impact (92.6%) communities [22]. Individuals exposed indirectly to wildfires were more likely to demonstrate maladaptive coping strategies and lower post-traumatic growth [27]. Toit et al. [35] found that adolescents exposed to bushfires did not show negative mental health outcomes in the long term. This could be associated with coping and resilience styles of young individuals, as seen after disasters [35]. These findings suggest that coping style, resilience, and mode of exposure play a critical role in shaping psychological outcomes following wildfires [12,22,27].

#### 3.2.2. Neurological and Cognitive Effects Associated with Wildfire Smoke Exposure

In addition to disaster-related psychological stress, exposure to wildfire smoke itself may influence neurological and cognitive outcomes through biological mechanisms associated with inhaled particulate matter. Wildfire smoke contains high concentrations of fine particulate matter (PM_2.5_) and other combustion-derived pollutants that can induce systemic inflammation, oxidative stress, and vascular dysfunction. Several epidemiological studies have therefore examined associations between wildfire-related PM_2.5_ exposure and neurological or mental health outcomes.

Wildfire-related PM_2.5_ exposure has been associated with adverse mental health outcomes in several epidemiological studies. Jung et al. [37] reported that wildfire-related PM_2.5_ exposure was associated with increased emergency department visits for mental health conditions, with a total of 86,609 visits linked to wildfire-related PM_2.5_ exposure during wildfire events. The study also observed delayed associations, with elevated emergency department visits for anxiety occurring approximately three days after exposure, and an increased risk of depression-related visits among women following wildfire PM_2.5_ exposure [37]. Similarly, Zhu et al. [38] found that wildfire-related PM_2.5_ exposure was positively associated with increased emergency department visits for anxiety disorders. These findings suggest that wildfire smoke exposure may contribute to short-term increases in anxiety-related health care utilization [37]. In a population-based study, Mirabelli et al. [39] examined the effects of prolonged smoke exposure and reported that exposure to heavy smoke for more than four weeks was associated with a 34% increased prevalence of feelings of nervousness, anxiety, or being on edge in the previous two weeks. Exposure to heavy and medium smoke was also associated with a 29% and 30% increased prevalence of difficulty controlling worry, respectively. In addition to mental health outcomes, cognitive effects have also been observed. Cleland et al. [11] reported that wildfire-related PM_2.5_ exposure was negatively associated with attention performance, with the strongest effects observed following short-term exposure. Reduced attention was detected within three hours of wildfire PM_2.5_ exposure, and medium to heavy smoke density was associated with significant declines in attention scores [11]. Sex differences were also noted, with male participants exhibiting stronger negative associations with heavy smoke exposure compared with females [11].

Collectively, evidence from human studies indicates that wildfire exposure is consistently associated with increased prevalence of depression, PTSD, anxiety, insomnia, and cognitive impairment. However, the independent contribution of wildfire-related PM_2.5_ to these above-mentioned outcomes remains difficult to isolate, as wildfire events often involve co-occurring stressors such as extreme heat, evacuation or displacement, socioeconomic vulnerability, and pre-existing mental health conditions.

### 3.3. Wildfires and Epigenetics

#### 3.3.1. Impact on Animals

Emerging preclinical evidence suggests that wildfire smoke exposure is associated with epigenetic and transcriptomic changes across species. Schuller et al. [16] used RNA sequencing of microdissected prefrontal cortex tissue from wildfire PM_2.5_-exposed male mice and identified 2862 differentially expressed genes (1396 upregulated; 1466 downregulated). One of the most significantly upregulated genes was vinculin (*Vcl*), which is involved in cytoskeletal organization and cell adhesion, and may be relevant to BBB function. Downregulated genes included pregnancy-specific glycoprotein 16 (*Psg16*) and β-1,3-galactosaminyltransferase polypeptide 1 (*B3galnt1*). Furthermore, pathway analyses suggested disruptions in serotonergic, cholinergic, and dopaminergic signaling networks, consistent with broad neuromodulatory effects [16].

Extending these findings beyond transcriptomic changes in the brain, Brown et al. [15] investigated the long-term epigenetic consequences of early-life wildfire smoke exposure in female rhesus macaques. Genome-wide DNA methylation analysis identified 3370 differentially methylated regions (DMRs) enriched in pathways involved in synaptogenesis, protein kinase A signaling, and immune function. Notably, many of these DMRs were located in bivalent chromatin regions, which play a key role in developmental gene regulation, suggesting potential long-lasting effects of early exposure [15]. Evidence for wildfire-related epigenetic effects is not limited to somatic tissues. Schuller et al. [14] reported that simulated wildfire smoke exposure in mice altered sperm DNA methylation patterns, with exposed animals showing distinct clustering from controls. Gene ontology analyses of DMR-associated genes highlighted developmental pathways, raising the possibility that wildfire smoke exposure may influence offspring health through germline epigenetic modifications [14].

Complementing these molecular findings, Vokina et al. [20] examined acute peat smoke exposure in Wistar rats and observed both behavioral and electrophysiological alterations. Exposed animals displayed increased anxiety-like behavior in the open-field test and changes in EEG rhythms, characterized by increased δ-rhythm in the right hemisphere and decreased beta-2 rhythm in the left hemisphere. Although spatial learning was not impaired, global DNA hypomethylation detected in blood cells suggested systemic epigenetic effects following smoke exposure [20].

#### 3.3.2. Impact on Humans

Evidence in humans remains limited, with only a small number of studies directly examining epigenetic responses to wildfire exposure. Goodrich et al. [40] investigated miRNA expression and DNA methylation in firefighters exposed to wildland–urban interface fires. Following the wildfire season, 65 miRNAs were differentially expressed compared with pre-season samples. Enrichment analyses indicated that these altered miRNAs mapped to gene sets associated with asthma, carcinoma, thyroid neoplasms, inflammation, and hepatocellular carcinoma. One miRNA, hsa-miR-518c-3p, was notably downregulated following exposure. In contrast, no significantly differentially methylated loci were identified between pre- and post-season samples in the DNA methylation analysis, suggesting that miRNA expression may be more sensitive to short-term wildfire exposure than site-specific DNA methylation changes [40]. Complementing these findings, Xu et al. [41] examined associations between wildfire-related PM_2.5_ exposure and global DNA methylation measures. The study found a strong association between wildfire-related PM_2.5_ and 7 measures of global DNA methylation, which decreased [41]. CpGs and DMRs associated with wildfire-related PM_2.5_ were different from non-wildfire-related PM_2.5_ [41]. 4 KEGG pathways were significantly augmented for CpGs and DMRs associated with wildfire-related PM_2.5_, whereas no KEGG pathway was significantly augmented for non-wildfire-related PM_2.5_ [41]. The genes impacted by wildfire-related PM_2.5_ were related to cancer, mental disorders, diabetes, obesity, asthma, and blood pressure [41]. These studies highlight the importance of exploring the effects of wildfire and air pollution on epigenetics, specifically DNA methylation and gene expression [40,41]. The findings suggest that a variety of genes and pathways are associated with the effects of wildfire and air pollution exposures [40,41].

Taken together with findings from animal models, emerging evidence suggests that wildfire smoke exposure may be associated with epigenetic and transcriptomic alterations across species. Experimental studies in animals demonstrate changes in brain gene expression, DNA methylation, and germline epigenetic profiles following wildfire smoke exposure, supporting potential mechanisms for long-term and possibly intergenerational effects. In human studies, wildfire-related particulate matter has been associated with epigenetic signatures that may differ from those observed with non-wildfire air pollution, suggesting that wildfire smoke represents a distinct environmental exposure with unique biological effects. However, the current human evidence remains limited. Most studies assess epigenetic alterations in peripheral tissues, such as blood or nasal epithelium, rather than directly examining brain-related mechanisms or neurological outcomes. In addition, available studies are largely cross-sectional and often involve relatively small sample sizes, which limits causal inference. Consequently, current findings should be interpreted as associational and hypothesis-generating, highlighting potential biological pathways linking wildfire exposure to neurological and mental health outcomes rather than demonstrating established mechanisms.

## 4. Discussion

This review synthesizes current evidence from experimental animal studies and human investigations examining the neurological, mental health, and epigenetic effects of wildfire exposure. Collectively, the available literature suggests that wildfire smoke can induce neuroinflammatory and metabolic changes in experimental models, while human populations exposed to wildfire events frequently report increased prevalence of mental health symptoms such as depression, anxiety, PTSD, and sleep disturbances. However, most human studies examining these outcomes are observational and are conducted in the context of wildfire disasters rather than controlled smoke exposure, which complicates the interpretation of the reported associations. An important challenge in interpreting the human literature is that wildfire events represent complex environmental disasters involving both toxicological smoke exposure and psychosocial stressors, including evacuation, displacement, and perceived threat to life. In addition, factors such as extreme heat, socioeconomic vulnerability, displacement, and pre-existing mental health conditions may act as potential confounders in epidemiological studies of wildfire exposure. As a result, many epidemiological studies capture the combined effects of these pathways, making it difficult to isolate the independent contribution of wildfire smoke exposure. Consequently, associations between wildfire-related PM_2.5_ exposure and mental health outcomes should be interpreted cautiously, as the specificity of smoke-driven effects remains uncertain.

Experimental animal studies provide important mechanistic insight into the potential neurological effects of wildfire smoke exposure. Inhaled particulate matter and combustion-derived pollutants may activate systemic inflammatory responses, promote oxidative stress, and alter vascular function, which in turn may influence brain physiology. Several animal studies reviewed here report neuroinflammatory responses, metabolic disruptions, and behavioral changes following exposure to biomass or wildfire-related smoke particles. Although these models provide valuable insight into potential biological mechanisms, it should be noted that exposure conditions in experimental studies often differ from those experienced by human populations during wildfire events. Consequently, caution is required when extrapolating these findings to human health outcomes.

An important conceptual consideration in this literature is the distinction between wildfire smoke exposure and wildfire disaster exposure. While inhaled particulate matter may contribute to neurological and cognitive effects through biological pathways such as inflammation and oxidative stress, wildfire disasters also generate substantial psychological stress due to evacuation, loss of property, community disruption, and perceived threat to life. Many studies reporting increased prevalence of PTSD, depression, and anxiety following wildfires therefore reflect the psychological consequences of disaster exposure rather than isolated toxicological effects of smoke inhalation. Future research integrating detailed environmental exposure assessment with mental health evaluation will be essential for disentangling these pathways.

### 4.1. Sex Differences

Accumulating evidence suggests that sex differences may influence mental health outcomes following wildfire exposure; however, findings across studies remain heterogeneous and context dependent. Some epidemiological studies report a higher prevalence of PTSD among females compared with males after wildfires [29]. Females have also been reported to show higher rates of diagnoses such as major depressive disorder and generalized anxiety disorder, whereas males may exhibit higher rates of high-risk alcohol consumption and nicotine dependence. In one study, at 18 months post-exposure, the one-month prevalence of probable PTSD among females was approximately double that observed among males. However, these patterns are not uniform across studies and may reflect differences in study design, population characteristics, and assessment methods. These observations suggest that sex-related differences in psychological responses, coping strategies, social roles, and health behaviors may contribute to variability in mental health trajectories following wildfire exposure [29].

Differences in risk perception and emotional processing may further explain these disparities. Lachlan et al. [42] reported that women perceived wildfires as posing a greater risk and experienced higher emotional stress prior to fire events compared with men. Such differences in threat perception and anticipatory stress may contribute to variations in psychological responses to wildfire events, although these associations may depend on contextual factors such as previous disaster experience, cultural context, and social support systems [29,42].

Preclinical studies provide additional insight into potential biological mechanisms underlying sex-related differences, although findings remain variable across experimental models. Wardhani et al. [19] reported that male mice exposed to wildfire smoke showed heightened proinflammatory cytokine expression, whereas female responses varied across brain regions. Importantly, ovariectomized females showed amplified cortical inflammation and reduced anti-inflammatory signaling in the hippocampus, highlighting the modulatory role of ovarian hormones in smoke-induced neuroinflammation. These findings suggest that hormonal status may influence susceptibility to wildfire-related neurological effects. Beyond inflammatory pathways, sex-dependent metabolic responses have also been observed. Female mice exhibited more pronounced lipidomic alterations following smoke exposure, suggesting that biological sex influences metabolic responses to wildfire pollutants [19]. In addition, paternal exposure studies have shown that offspring—particularly females—exhibit greater anxiety-like behavior and cognitive impairments following biomass smoke exposure [6].

Taken together, these experimental findings suggest that biological sex and hormonal status may influence responses to wildfire smoke exposure, although the relevance of these mechanisms in human populations remains to be fully clarified.

### 4.2. Vulnerable Populations

Certain populations appear to be disproportionately affected by the mental health and potential epigenetic consequences of wildfire exposure, including children, pregnant individuals, those of lower socioeconomic status, immunocompromised individuals, and Indigenous communities. Developmental vulnerability is particularly evident in children, with younger children exhibiting more severe psychological symptoms following wildfire exposure than older youth [30]. Children exposed to wildfires have shown increased prevalence and severity of depression, anxiety, PTSD, suicidal ideation, and tobacco use [10,12], raising concerns about long-term impacts on psychological development.

Pregnant individuals represent another population of concern. Although direct evidence on wildfire smoke exposure during gestation remains limited [43], pregnant people may be particularly susceptible due to wildfire-related respiratory and cardiovascular stressors. Proposed biological mechanisms include oxidative stress, endothelial dysfunction, metabolic disruption, DNA damage, and epigenetic alterations, all of which have been linked to adverse pregnancy outcomes such as preterm birth, fetal growth restriction, and pregnancy loss. While the placenta may act as a partial barrier to environmental exposures, evidence suggests that particulate matter can penetrate this barrier, potentially increasing fetal vulnerability. These findings highlight the need for targeted research on gestational wildfire exposure and epigenetic programming during early development [43].

Socioeconomic disadvantages further exacerbate wildfire-related health risks. Communities with fewer resources to mitigate smoke exposure experience more frequent and prolonged exposure to wildfire smoke [44]. Lower socioeconomic status has also been associated with increased risk of adverse cardiovascular outcomes following wildfire exposure, including out-of-hospital cardiac arrest [45]. Immunocompromised individuals may likewise face heightened vulnerability due to reduced physiological resilience to smoke-related inflammatory and oxidative stressors [46].

Indigenous communities worldwide face unique and compounded risks due to structural inequities and the cultural significance of land. Damage to traditional lands and ecosystems can threaten cultural identity and intensify psychological distress. Macleod et al. [47] reported that Indigenous Australians exposed to the 2019–2020 bushfires experienced higher levels of depression, anxiety, and PTSD compared with non-Indigenous Australians, yet also demonstrated higher resilience and well-being. These findings underscore the importance of incorporating Indigenous perspectives and culturally grounded approaches into wildfire preparedness, response, and mental health care.

Overall, vulnerable populations should be prioritized in both research and public health interventions addressing wildfire-related mental health and epigenetic risks.

### 4.3. Epigenetics and Mental Health

Growing evidence suggests that environmental exposures, including air pollution and wildfire smoke, may influence mental health through epigenetic mechanisms. Epigenetic regulation—such as DNA methylation, histone modification, and non-coding RNA signaling—can alter gene expression without changing the underlying DNA sequence and may respond dynamically to environmental stressors. As a result, epigenetic alterations associated with wildfire exposure may represent a potential biological pathway contributing to the development or exacerbation of mental health disorders.

Evidence from non-wildfire contexts provides important support for this hypothesis. Several studies have identified associations between DNA methylation patterns and mental health outcomes. For example, Martinez et al. [48] reported increased DNA hypermethylation in individuals experiencing anxiety and depression during pregnancy, particularly in promoter regions. Similarly, Taylor et al. [49] identified differentially methylated positions and regions associated with depressive symptoms in Black women, implicating genes involved in neurological and immune-related pathways. Katrinli et al. [50] further reported differential methylation patterns associated with PTSD among individuals of low socioeconomic status. These findings suggest that epigenetic regulation may play an important role in the biological embedding of psychological stress and mental health vulnerability.

Emerging wildfire-specific research provides preliminary evidence supporting this possibility. Experimental studies have demonstrated alterations in gene expression profiles and DNA methylation patterns following wildfire smoke exposure in animal models. Limited human studies have also reported associations between wildfire exposure and epigenetic markers measured in peripheral tissues such as blood or nasal epithelium. These experimental findings provide mechanistic insights that may help interpret epidemiological observations linking wildfire exposure to neurological and mental health outcomes. In particular, molecular responses observed in animal models—including altered gene regulation and inflammatory signaling—represent plausible biological pathways through which wildfire smoke exposure could influence psychological and cognitive outcomes in exposed populations.

However, the current human evidence remains sparse. Most studies assess epigenetic changes in peripheral tissues rather than brain tissue, and few directly link epigenetic alterations to neurological or mental health outcomes in wildfire-exposed populations. In addition, many existing studies involve relatively small sample sizes or indirect exposure assessments, limiting causal inference. Consequently, current findings should be interpreted as associational and hypothesis-generating rather than demonstrating established causal mechanisms.

Despite these limitations, the emerging evidence highlights the potential importance of epigenetic pathways in understanding the long-term health effects of wildfire exposure. Future research integrating detailed wildfire exposure assessment, longitudinal cohort designs, epigenetic profiling, and mental health outcomes within the same populations will be critical for clarifying whether epigenetic alterations contribute to wildfire-related neurological and psychological effects.

### 4.4. Transgenerational Effects

Beyond immediate health outcomes, wildfire smoke exposure raises concerns about potential transgenerational effects mediated by epigenetic mechanisms. Animal studies provide preliminary evidence that prenatal and preconception exposures may induce heritable molecular changes. Brown et al. [15] reported that early-life wildfire smoke exposure in rhesus macaques resulted in thousands of differentially methylated regions enriched for immune and neuronal pathways, despite minimal baseline transcriptional changes. This pattern suggests latent epigenetic priming that may increase vulnerability under subsequent stressors. Similarly, Schuller et al. [14] suggested that simulated wildfire smoke exposure induced widespread hypermethylation in mouse sperm DNA, implicating potential germline transmission of smoke-related epigenetic modifications. Rodent studies further support this possibility, with peat smoke exposure associated with DNA methylation changes, anxiety-like behavior, and altered motor activity [20]. Prolonged wildfire smoke exposure has also been associated with global hypermethylation in murine sperm, consistent with broader evidence linking abnormal sperm DNA methylation to impaired reproductive outcomes [51].

However, the persistence of exposure-induced epigenetic marks across generations remains uncertain. DNA methylation patterns undergo extensive reprogramming during early development, particularly in primordial germ cells and shortly after fertilization [52]. These processes may erase environmentally induced methylation marks, including those related to wildfire exposure. Consequently, future studies should investigate whether wildfire-induced epigenetic alterations can evade reprogramming and contribute to transgenerational health effects in both animal models and human populations.

### 4.5. Limitations of the Review

Several limitations of the current review should be acknowledged.

First, this study was conducted as a narrative review, which aims to synthesize and contextualize existing literature rather than perform a formal systematic evaluation of study quality. Although a structured search strategy was used to identify relevant studies, the narrative approach does not include a formal risk-of-bias assessment and may therefore be subject to selection bias.

Second, the literature on wildfire exposure and mental health outcomes is heterogeneous, with studies differing in exposure assessment, outcome measurement, and study design. Most human studies examining these outcomes are observational and are conducted in the context of wildfire disasters rather than controlled smoke exposure, which complicates the interpretation of reported associations. Wildfire events represent complex environmental disasters involving both toxicological smoke exposure and psychosocial stressors, including evacuation, displacement, and perceived threat to life. In addition, factors such as extreme heat, socioeconomic vulnerability, displacement, and pre-existing mental health conditions may be confounders in epidemiological studies of wildfire exposure. Consequently, many studies capture the combined effects of these pathways, making it difficult to isolate the independent contribution of wildfire smoke exposure. As a result, associations between wildfire-related PM_2.5_ exposure and mental health outcomes should be interpreted cautiously, as the specificity of smoke-driven effects remains uncertain.

Third, the evidence linking wildfire exposure to epigenetic changes in human populations remains limited, with relatively few studies available and most relying on peripheral tissue samples rather than brain tissue. This limits the ability to directly connect epigenetic alterations with neurological or mental health outcomes.

Finally, this review did not specifically focus on dose–response relationships between wildfire smoke exposure and neurological or mental health outcomes. The present review, therefore, does not attempt to define exposure thresholds or quantify exposure–response relationships. Future research integrating precise exposure assessment, longitudinal study designs, and multi-omics approaches will be essential for clarifying how varying levels and durations of wildfire smoke exposure influence neurological and mental health risks.

### 4.6. Future Research

Despite growing recognition of the neurological and mental health impacts of wildfire exposure, substantial gaps remain in understanding the underlying biological mechanisms, particularly the role of epigenetic regulation. While experimental animal studies have begun to characterize epigenetic alterations following wildfire smoke exposure, human evidence remains limited. Future research should therefore prioritize investigations of epigenetic changes in human populations exposed to wildfire events, particularly studies that integrate molecular data with mental health outcomes.

Longitudinal studies will be especially important for clarifying the temporal dynamics of both mental health outcomes and epigenetic alterations following wildfire exposure. Prospective cohort designs can help determine whether epigenetic changes resolve, persist, or evolve and how these patterns relate to trajectories of psychological well-being. At present, there is a lack of longitudinal research simultaneously examining wildfire exposure, epigenetic regulation, and mental health outcomes. In addition, greater standardization in the assessment of mental health outcomes is needed, as variation in measurement approaches—including differences in self-reported measures and standardized questionnaires—can limit comparability across studies.

Improved exposure characterization will also be essential for advancing this field. Future research should aim to disentangle the health effects of specific wildfire-derived pollutants, including particulate matter (PM_2.5_), polycyclic aromatic hydrocarbons (PAHs), and heavy metals. Integrating detailed environmental exposure assessment with molecular analyses—such as epigenomic, transcriptomic, or metabolomic profiling—may help clarify biological pathways linking wildfire smoke exposure to neurological and mental health outcomes. Greater attention should also be given to vulnerable populations, including children, pregnant individuals, and communities experiencing repeated wildfire exposure.

Finally, research evaluating potential mitigation strategies is needed to inform public health responses to wildfire smoke exposure. Community-level interventions such as improved air-quality monitoring, early warning systems, and the establishment of clean-air shelters may help reduce exposure during wildfire events. At the individual level, strategies including indoor air filtration systems, high-efficiency particulate air (HEPA) filters, and limiting outdoor activities during periods of elevated smoke may also reduce exposure to wildfire-related pollutants. However, further research is needed to determine whether these interventions can effectively mitigate potential neurological or mental health consequences associated with wildfire smoke exposure.

## 5. Conclusions

The studies reviewed highlight growing evidence that wildfire exposure may influence neurological and mental health outcomes through multiple pathways. Experimental animal studies provide strong mechanistic signals showing that wildfire smoke exposure can induce neuroinflammation, alterations in brain gene expression, and epigenetic modifications. In human populations, wildfire disasters are consistently associated with increased mental health burden, including elevated rates of depression, anxiety, post-traumatic stress disorder (PTSD), and sleep disturbances. However, many of these studies reflect the combined impact of environmental smoke exposure and disaster-related psychosocial stressors.

Evidence specifically linking wildfire smoke or wildfire-related PM_2.5_ exposure to neurological or cognitive outcomes in humans remains more limited. Similarly, although emerging studies suggest that wildfire exposure may be associated with epigenetic alterations, the current human evidence is sparse and primarily based on peripheral tissues rather than direct assessment of brain mechanisms or mental health outcomes. As a result, proposed epigenetic pathways linking wildfire exposure to neurological or psychological outcomes should currently be interpreted as hypothesis-generating rather than established causal mechanisms.

As wildfires continue to increase in frequency and intensity worldwide, further research integrating precise exposure assessment, longitudinal study designs, and molecular profiling will be essential for clarifying how wildfire smoke and disaster-related stress may contribute to neurological and mental health outcomes. Improving understanding of these pathways will help inform public health preparedness strategies and identify potential approaches to mitigate the health impacts of future wildfire events.

## Data Availability

No new data were created or analyzed in this study.

## Abbreviations

The following abbreviations are used in this manuscript:

BBB	Blood-Brain Barrier
B3galnt1	β-1,3-galactosaminyltransferase polypeptide 1
CA3	Cornu Ammonis region 3
CD31	Platelet endothelial cell adhesion molecule-1 (PECAM-1)
CNS	Central Nervous System
CO	Carbon Monoxide
CpG	Cytosine-Phosphate-Guanine
DMR	Differentially Methylated Regions
GAD	Generalized Anxiety Disorder
GFAP	Glial Fibrillary Acidic Protein
HPA	Hypothalamic-Pituitary-Adrenal axis
Iba-1	Ionized Calcium-Binding Adapter Molecule 1
iNOS	Inducible Nitric Oxide Synthase
miRNA	MicroRNA
MDD	Major Depressive Disorder
MBP	Myelin Basic Protein
NAD^+^	Nicotinamide Adenine Dinucleotide
NO_X_	Nitrogen oxides
O _3_	Ozone
PAHs	Polycyclic aromatic hydrocarbons
PM	Particulate matter
Psg16	Pregnancy-specific glycoprotein 16
PTSD	Post-Traumatic Stress Disorder
RNMN	Resveratrol plus Nicotinamide Mononucleotide
RNDQ	Resveratrol + Nicotinamide Mononucleotide plus Dasatinib + Quercetin
Vcl	Vinculin
VOC	Volatile organic compounds
